# Inhalation Toxicology of Vaping Products and Implications for Pulmonary Health

**DOI:** 10.3390/ijms21103495

**Published:** 2020-05-15

**Authors:** Hussein Traboulsi, Mathew Cherian, Mira Abou Rjeili, Matthew Preteroti, Jean Bourbeau, Benjamin M. Smith, David H. Eidelman, Carolyn J. Baglole

**Affiliations:** 1 Research Institute of the McGill University Health Centre, Montreal, QC H4A 3J1, Canada; Hussein.traboulsi@mail.mcgill.ca (H.T.); mira.abourjeili@mail.mcgill.ca (M.A.R.); matthew.preteroti@mail.mcgill.ca (M.P.); jean.bourbeau@mcgill.ca (J.B.); benjamin.m.smith@mcgill.ca (B.M.S.); 2 Department of Medicine, McGill University, Montreal, QC H4A 3J1, Canada; mathew.cherian@mail.mcgill.ca (M.C.); david.h.eidelman@mcgill.ca (D.H.E.); 3 Respiratory Epidemiology and Clinical Research Unit, McGill University Health Centre, Montreal, QC H4A 3J1, Canada; 4 Department of Pathology, McGill University, Montreal, QC H3A 2B4, Canada; 5 Department of Pharmacology and Therapeutics, McGill University, Montreal, QC H3G 1Y6, Canada

**Keywords:** e-cigarettes, cannabis, vaping, respiratory, inflammation, cannabinoid, lung injury, EVALI, lipoid pneumonia, vitamin E acetate

## Abstract

E-cigarettes have a liquid that may contain flavors, solvents, and nicotine. Heating this liquid generates an aerosol that is inhaled into the lungs in a process commonly referred to as vaping. E-cigarette devices can also contain cannabis-based products including tetrahydrocannabinol (THC), the psychoactive component of cannabis (marijuana). E-cigarette use has rapidly increased among current and former smokers as well as youth who have never smoked. The long-term health effects are unknown, and emerging preclinical and clinical studies suggest that e-cigarettes may not be harmless and can cause cellular alterations analogous to traditional tobacco smoke. Here, we review the historical context and the components of e-cigarettes and discuss toxicological similarities and differences between cigarette smoke and e-cigarette aerosol, with specific reference to adverse respiratory outcomes. Finally, we outline possible clinical disorders associated with vaping on pulmonary health and the recent escalation of acute lung injuries, which led to the declaration of the vaping product use-associated lung injury (EVALI) outbreak. It is clear there is much about vaping that is not understood. Consequently, until more is known about the health effects of vaping, individual factors that need to be taken into consideration include age, current and prior use of combustible tobacco products, and whether the user has preexisting lung conditions such as asthma and chronic obstructive pulmonary disease (COPD).

## 1. Introduction

Tobacco smoking remains the leading cause of preventable death worldwide and is the single greatest risk factor for developing chronic diseases such as chronic obstructive pulmonary disease (COPD) and lung cancer. Despite knowledge about the dangers of tobacco and tobacco constituents, we are seeing a surge in the use of emerging tobacco-based products such as heat-not-burn products (IQOS) and e-cigarettes, which are also called vape devices or more formally as electronic nicotine delivery systems (ENDS). E-cigarettes consist of a rechargeable battery; an atomizer (or heating element/coil); and a liquid that contains a solvent (usually propylene glycol (PG) and vegetable glycerin (VG)), nicotine, and various additives including flavors; e-cigarettes do not contain tobacco but function to deliver nicotine to the brain. Vaping is the act of inhaling into the lungs the vapor or aerosol that is produced by an e-cigarette. It is designed to simulate the act of smoking but without tobacco combustion, the latter of which releases thousands of toxicants including many carcinogens. Because, empirically, there are lower quantities of toxicants associated with combustion, it is believed that these products can be used as a cessation tool for heavy smokers and thus as a way towards greater health improvement [1]. Even if these are more efficacious for certain individuals in comparison to nicotine replacement therapies (NRT), there is little scientific evidence in support of this claim. Moreover, these products have greatly increased in popularity amongst teens and young adults in the United States and Canada [2,3]. Although vaping has been generally recognized as safer than tobacco smoke, in reality, the long-term health effects of e-cigarette use are not known. While the relative newness of e-cigarette use makes discussion about long-term effects difficult, there is a growing body of literature that e-cigarette use may lead to effects that are not dissimilar to cigarette smoking at a cellular, clinical, and population level.

Additionally, there is now a growing number of health concerns from vaping, particularly over a recent outbreak of severe lung disease (associated with counterfeit vaping products) that has been coined “vaping product-associated lung illness (EVALI) or “vape lung” [4]. Largely confined to the Unites States, over 2000 cases have been reported, and as of January 2020, the Centers for Disease Control and Prevention (CDC) confirmed 60 deaths from EVALI [4,5,6]. Cases are now being reported in Canada but not in European countries. EVALI is generally associated with adverse respiratory outcomes including shortness of breath, cough, and hypoxemia [7]. Patients (majority male) also typically reported using tetrahydrocannabinol (THC)-containing products [5]. THC is the psychoactive cannabinoid from *Cannabis sativa*. Public health investigations have identified that the likely etiologic agent in EVALI is vitamin E acetate, which is used as a thickening agent in THC-containing vape products. Thus, the use of e-cigarettes (1) lacks long-term data on the safety of its chronic use; (2) is associated with an outbreak of acute vaping associated lung injury in North America, and (3) raises considerable concern of creating a new generation of young adults addicted to nicotine. Given the raising prevalence of e-cigarette use in today’s society and emerging evidence of respiratory consequences [8], this review will focus on the inhalation toxicology of vaping products, factors affecting the chemical composition of the aerosol, and their perturbation of cellular and molecular processes than may lead to respiratory disease.

## 2. Historical Context of Cigarette Use and Evidence for Disease Induction

When evaluating the adverse effects associated with acute and chronic exposure to e-cigarette aerosols, reflexion on events that led to the rise in cigarette popularity in the last century is warranted. Tobacco consumption in the early 1800s was largely through the use of snuff, chewing tobacco, or cigars [9]. Cigarettes did not gain attention until the early 1900s. The rise in popularity of cigarettes was due to several key events [10], one of which was the ways in which tobacco was cured. Previous methods to cure tobacco leaves included fire curing, air curing, and sun curing, all of which created a product that was harsh on the lungs when inhaled, thus making smoking a less desirable consumption method [11]. During the 1830s in North Carolina, flue curing was adapted [12]. Here, tobacco leaves are hung on tier-poles that was fed from external fire boxes which slowly increases the temperature indirectly [13]. This curing method increases the sugar content of the tobacco leaves, which makes the tobacco product much more tolerable when inhaled as smoke [12,13]. Another key hallmark in the rising cigarette popularity was the introduction of safety matches in the 1850s [11]. The next event that led to a rise in cigarette popularity was the invention of an automated cigarette rolling machine by James Bonsack in 1881. This machine could roll 200 cigarettes per minute [14]. Current-day cigarette rolling machines produce in excess of 273 cigarettes every second. American cigarette company Phillip Morris produces in excess of 730 million cigarettes every day [15]. As cigarette smoking was gaining popularity, two notable events played significant roles in facilitating their mass consumption: war rations and mass advertising [11]. Predominantly during World War I, soldiers would have cartons of cigarettes included in their rations. Cigarettes were also the most widely advertised consumer product of the time. Together, these events led to the peak prevalence of cigarette smoking, with approximately 67% of American men smoking cigarettes in the 1950s [11].

Despite the rapid rise in cigarette use, the health consequences of tobacco smoke were largely unknown. Even before the popular use of cigarettes, one of the first observations of possible adverse health consequences of tobacco use came in 1761, when John Hill associated the use of tobacco snuff as the cause of nasal polyps [16]. Other links between tobacco and adverse health effects emerged in the early 1900s when the German physician Hermann Rottmann noticed a correlation between women who worked at tobacco factories and an increased incidence of lung cancer, which he speculated may be the result of inhaled tobacco dust [17]. It was not until the 1950s that there was an experimental link between tobacco and cancer with a publication led by Ernst Wynder showing that tobacco tars, when applied to the dorsal area of mice, caused tumors [18]. A combination of experimental and epidemiological association led to the US Surgeon General declaration in 1964 that smoking cigarettes is related to cancer [19]. Today, tobacco smoking is known to cause a wide range of diseases with high mortality. This includes the two most common lung diseases: COPD, which causes an estimated 3.17 million deaths worldwide [20], and lung cancer, contributing to 1.76 million deaths in 2018 alone [21].

## 3. E-Cigarette Use Patterns and Their Utility as a Smoking Cessation Aid

With increasing awareness about the health consequences of tobacco smoking came the desire to create a means to replicate smoking. The first of these was a patent filed in 1963 by Herbert A. Gilbert for a smokeless nontobacco cigarette that resembles the modern e-cigarette [22]. For reasons that likely relate to the popularity of tobacco cigarette use during this time, the device was not brought to the market. It was not until 2003 when Hon Lik, a pharmacist, created a device that could vaporize a forced stream of nicotine as an alternative to classic cigarettes [14], with the rationale of creating a smoking cessation device [23,24,25]. E-cigarettes were subsequently introduced to the market in the United States in 2007 and have quickly gained in popularity, particularly among youth. The 2019 American National Youth Tobacco Survey (NYTS) revealed that 27.5% of high school students and 10.5% of middle school students were current users (defined as use in the past 30 days) of e-cigarettes [14,25]. In Canada, the 2017 Canadian Drug and Tobacco Survey showed that 23% of youth (ages 15–19) and 29% of young adults (ages 20–24) have tried e-cigarettes [25]. A national cross-sectional survey looking at the prevalence of vaping among adolescents (ages 16–19) in Canada, United States, and England in 2017 and 2018 (after the implementation of new regulations) showed that, in Canada in 2017, 29.3% of adolescents had tried e-cigarettes and 8.4% had reported use in the past 30 days [25]. In 2018, that number rose to 37% for ever-use and 14.6% for use in the past 30 days. The e-cigarette market continues to expand as the number of users rise. North America is the biggest market, with value of approximately US$ 5.7 billion in 2018 [26].

Common reasons for using an e-cigarette include liking the flavors, wanting to try something cool and new, and using them as a tool for smoking cessation [27,28]. Multiple surveys demonstrate that most e-cigarette users believe not only that e-cigarettes are less harmful than classic tobacco cigarettes but also that these devices may aid them to quit smoking traditional cigarettes. The evidence supporting a role for e-cigarettes in smoking cessation/reduction has not been straightforward. One study of 15 individuals, lead by a popular manufacturer of e-cigarettes, observed that the urge to smoke was reduced by e-cigarettes at plasma levels of nicotine that were lower than obtained with conventional cigarettes [29]. Another prospective 6-month study of 40 smokers with no intention of quitting found a sustained 50% reduction in cigarettes/day at week 24 in 13/40 participants and a sustained 80% reduction in 5/40 participants. No serious adverse events were reported during the 6 months, but 13/40 participants were lost to follow-up. However, no comparisons to other smoking cessation tools or to placebo was made [24]. Another year-long trial by the same group randomized 300 smokers to one of three arms: a popular e-cigarette model with 7.2 mg nicotine cartridges for 12 weeks; the same e-cigarette model with 7.2 mg nicotine cartridges for 6 weeks followed by 5.4 mg cartridges for another 6 weeks; or no nicotine cartridges for 12 weeks. Smoking reduction (≥50%) and smoking cessation across the three arms were documented in 10.3% and 8.7% of 183/300 participants available for follow-up 52 weeks later. However, no differences were observed between the three arms, including the no nicotine arm [30]. While e-cigarettes may be effective smoking cessation devices, the abstinence rates are considerably lower than those observed in studies using common smoking cessation therapies. For instance, Varenicline is a widely prescribed smoking cessation therapy, with smoking abstinence rates ranging from 22–35% at 1-year follow-up [31]. Among 886 participants randomized to either nicotine replacement products or e-cigarettes, complete abstinence at the 1-year mark was 9.9% in the nicotine replacement group and 18.0% in the e-cigarette group. Nonetheless, the continued use of and dependence on nicotine and the creation of dual users were issues in this trial. Among participants with 1-year abstinence, 80% were still using e-cigarettes in the e-cigarette group and 9% were still using nicotine replacement in the nicotine replacement group 52 weeks later. Additionally, while 18% of the e-cigarette users achieved complete abstinence, 25% (110/438) became dual users of e-cigarettes and conventional cigarettes [1].

Guidance on e-cigarette use has been mixed. Public Health England concludes that e-cigarettes, alone or in combination with other methods, appear useful in smoking reduction in the short term [32]. In the United States, the 2020 US. Surgeon General Report on Smoking Cessation concludes that there is not adequate evidence at this time to recommend e-cigarettes for smoking cessation [33]. Undoubtedly, long-term and comparative studies evaluating e-cigarettes against commonly used smoking cessation therapies as well as studies on the long-term safety of e-cigarettes are needed prior to conclusively establishing their utility as effective smoking cessation tools. Although research is divided on whether e-cigarettes can be considered a useful smoking cessation device, those unable to quit smoking might be better off using e-cigarettes containing nicotine over the long-term rather than continuing to smoke regular cigarettes, as completely switching from tobacco cigarettes could reduce exposure to many toxic and cancer-causing chemicals found in cigarette smoke. However, the lack of clinical data and long-term observational studies prevent us from being able to make any definitive conclusion.

## 4. E-Cigarette Components

An e-cigarette is a simple device that consists of a battery, atomizer, and a fluid reservoir that contains the e-liquid. While these are common components of all e-cigarettes, there are approximately 460 e-cigarettes brands currently available [34]. In addition, since their initial introduction in the market in 2007, there are now four generations of e-cigarettes. The first generation is called the “cig-a-like” that were designed to look and feel like a traditional cigarette. The second generation is known as “clearomizers” that has a higher volume fluid reservoir than the cig-a-like and where the fluid can be refilled. Third generation e-cigarettes are called “Mods” that have batteries which allow the consumer to vary the power. Finally, fourth generation e-cigarettes—the Pod or Pod-Mods—are the most recent type to enter the market; these have temperature control, variable voltage, and lower electrical resistance which increases the aerosol yield [35]. To produce an aerosol, an e-cigarette user takes a puff through the mouthpiece which activates an air-flow sensor, causing the atomizer to heat the e-liquid and to form an aerosol for inhalation to the lungs. Instead of an air flow sensor, some types of e-cigarettes use a push-button to activate the battery and to heat the atomizer [36,37]. With this latter style of device, the temperature that heats the liquid can be quite variable between devices and can reach 250 °C to create the aerosol [38]. Many of the features of an e-cigarette (e.g., temperature), along with composition of the e-liquid, can influence the generation of chemicals and particulate matter (PM) with the potential to produce adverse health effects.

## 5. E-liquids

E-liquids generally contain water, flavors, solvents, and nicotine. However, previous reports have identified more than 60 compounds in e-liquids, including compounds not specified by the manufacturer [10,31]. Although nicotine is a key component for many users, particularly those where smoking cessation is the primary goal, e-cigarettes are increasingly being used as a tool to inhale cannabis products, such as those which contain THC, the primary psychoactive cannabinoid produced by *C. sativa*. In the aerosol that is generated, additional chemicals are detected in both nicotine- and cannabis-based e-liquids. However, vaping cannabis-based products could be placed into a separate category from non-cannabis (or non-THC)-based e-liquids as their subsequent inhalation may cause unique patterns of lung injury. Indeed, more than 80% of hospitalized EVALI patients reported using THC-containing e-cigarettes and most often from illegal source called Dank Vape [39] (detailed in Section 8). Below, we outline the components of e-cigarette liquids and their chemical composition before and upon vaporization that may impact pulmonary health.

### 5.1. Solvents

Solvents are used to dissolve the flavors and nicotine, with PG and VG being two of the primary solvents for nicotine-containing products [40]. Many consumer products contain PG and VG, and both are generally recognized as safe (GRAS) by the Food and Drug Administration (FDA) to ingest [41], but their effects on the respiratory system are largely unknown. In addition to PG and VG, traces of other solvents are found in e-cigarettes, including ethylene glycol, toluene, and 1,3-Propanediol [42]. Ethylene glycol is an odorless, clear, and viscous liquid that is commonly used as a solvent in industries and as antifreeze in cooling and heating systems. While the health consequences of long-term exposure to ethylene glycol or to the other residual solvents from e-cigarettes have not been investigated, ethylene glycol is a respiratory irritant and may be associated with greater toxicity compared with conventionally used VG and PG [43]. In addition, polyethylene glycol 400 (PEG 400), medium chain triglycerides (MCT), and vitamin E acetate are three agents that are commonly added to cannabis-based vaping products. PEG 400 is a low-molecular-weight grade of PEG that is widely used in cosmetics products and pharmaceutical formulations as a solvent/lubricant due to its low oral and dermal toxicity. MCT is a fatty acid derived from coconut or palm that is often ingested as food or as a nutritional supplement. Vitamin E acetate is used in THC-containing vaping products and has been associated with EVALI cases [44]. Similar to PG and VG, both PEG 400 and MCT are recognized by the FDA as GRAS [45]. However, the potential health effects of inhaling aerosols containing these compounds have not been investigated.

### 5.2. Flavorings

There are more than 8000 unique e-liquid flavors available in the market [46], with tobacco, menthol/mint, and fruit flavors being the ones most preferred by consumers. Sweet flavors are particularly appealing to youth. Many e-cigarette products contain more than one flavoring chemical (average is approximately 6), and those with sweet flavors contain more chemicals compared with tobacco and menthol flavored liquids [47]. Many of the flavors used in e-cigarettes are considered GRAS to consume orally, but it is important to recognize that such ingredients have not been safety-tested for inhalation and that some flavor chemicals are in fact toxicants [48]. For example, saccharides are used as sweetening flavors that thermally degrade to furans and aldehydes [49]. Benzaldehyde, a chemical that is used in fruity flavors such as cherry, is present in 75% of 145 e-cigarette refill fluids [50]. While many concerns have been raised regarding the role of flavors to promoting nicotine dependency among youth, the role of flavoring agents in the etiology of certain respiratory illness may present a more acute concern. Another common flavoring agent is cinnamaldehyde, being present in 51% of the products sampled [48], and there is evidence that cinnamaldehyde is cytotoxic [51]. In fact, ten cinnamon flavored e-liquids from different brands demonstrated cytotoxicity in a dose-dependent manner [52]. One of the limitations in this study was the use of the e-liquid to evaluate cytotoxicity, not the resultant aerosol. In a follow-up study, investigators exposed human bronchial epithelial cells to aerosolized JUUL products containing various flavors using a puff regime of three puffs/min, with 55 mL puff volume over 22 min for a total of 66 puffs [46]; this regime is similar to human e-cigarette puff topography but with higher puff numbers [53]. Overall, exposure to aerosolized JUUL flavor pods induced a moderate inflammatory response and altered epithelial barrier function [46]. Variations in exposure regimes—some of which do not replicate human exposures—and the dosage make extrapolation of in vitro evidence difficult, and identification as to which constituents are responsible for many of the observed adverse effects unclear.

### 5.3. Nicotine

One of the main ingredients in e-liquids is nicotine. Nicotine is the principal alkaloid of tobacco that occurs throughout the tobacco plant, acting as a botanical insecticide. Nicotine was named after the French Ambassador to Portugal Jean Nicot, who introduced tobacco seeds in Paris in 1550, and is the main addictive ingredient in tobacco. Nicotine is a weak base, and in its ionized state, such as in acidic environments, nicotine does not rapidly cross membranes. The pH of smoke from flue-cured tobacco found in most cigarettes is acidic (pH 5.5–6.0) [54]. When tobacco smoke reaches the small airways and alveoli of the lung, nicotine is rapidly absorbed, likely because dissolution of nicotine occurs in the fluid (pH 7.4) in the human lung, which facilitates transfer across membranes. Nicotine binds the nicotinic acetylcholine receptors (nAChRs) present in the central and peripheral nervous systems as well as various other organs. nAChRs are ligand-gated ion channels that trigger the release of neurotransmitters including dopamine that elicits rewarding behavior. These receptors are also expressed by endothelial, pulmonary epithelial, immune, and muscle cells [55,56,57]. Besides contributing to addictive properties of tobacco smoke, nicotine may contribute to cardiovascular disease in smokers [58] and may impact the function of respiratory and gastrointestinal systems.

The average tobacco rod of a traditional cigarette contains 10–14 mg of nicotine [59] and delivers approximately 2 mg of nicotine to the user. Nicotine content varies in e-liquids, with concentrations ranging between 16 and 24 mg/mL. Although nicotine can be synthesized from other chemicals, this process is quite expensive. Thus, the vast majority of commercially-available nicotine is extracted from tobacco plants [60]. In June 2015, an ultraportable e-cigarette device called JUUL was introduced to the market. One JUUL pod contains the same amount of nicotine as up to two packs of cigarettes [61]. Some of these newer e-cigarettes (e.g., JUUL) contain a nicotine base and a weak organic acid (e.g., benzoic acid) that forms a nicotine salt once the device is activated [62]. Nicotine salts are more tolerable to the lungs when inhaled, leading to delivery of higher concentrations of nicotine [63]. It is possible that users of these products would get even more nicotine than from a traditional tobacco cigarette. For this reason as well as their small compact design, there has been a dramatic increase the use of JUUL, particularly by youth, since their introduction in the market. Currently, JUUL is the most popular brand of e-cigarettes in North America, accounting for over than 70% of the US e-cigarette market [64,65].

### 5.4. Cannabinoids

*C. sativa* is an annual dieocious plant commonly known as marijuana. Cannabis contains over 100 secondary metabolites known as cannabinoids [66], including THC and cannabidiol (CBD), the latter being the most abundant nonpsychoactive cannabinoid. Cannabis is the second most- smoked product after tobacco [67]. Like tobacco smoke, burning cannabis produces hundreds of chemicals, including carcinogens and other toxicants [68]. Alternative methods of cannabis use are becoming popular, including vaporization (“cannavaping”), a technique that heats the dried plant without igniting it. In addition, THC and CBD oil/liquid can now be vaporized for inhalation (analogous to an e-cigarette except that THC or CBD replaces nicotine). Owing to issues of legality, there is a dearth of research investigating risks associated with exposure to cannabinoids by vaping. While adults are being admitted to hospitals due to suspected exposure to cannabis-derived vaping products [69], the overall health effect of cannabis vaping is largely unknown.

## 6. E-Cigarette Toxicology

Tobacco smoke is a complex aerosol which includes condensed liquid droplets (the particulate fraction or tar) suspended in a mixture of volatile and semivolatile compounds and combustion gases (the gas fraction). The gas phase of cigarette smoke includes nitrogen (N_2_), oxygen (O_2_), carbon dioxide (CO_2_), CO, acetaldehyde, methane, hydrogen cyanide (HCN), nitric acid, acetone, acrolein, ammonia, methanol, hydrogen sulfide (H_2_S), hydrocarbons, gas phase nitrosamines, and carbonyl compounds. Constituents in the particulate phase include carboxylic acids, phenols, water, nicotine, terpenoids, tobacco-specific nitrosamines (TSNAs), polycyclic aromatic hydrocarbons (PAHs), and catechols. Studies are now emerging that the aerosol generated from e-cigarettes also contains many of these same toxic compounds and varying size distribution of PM not unlike that of cigarette smoke. However, many of these compounds—and the associated PM—are present in e-cigarette aerosols at lower amounts than conventional cigarette smoke. Another consideration is the pH of the resultant aerosol, which will determine the fraction of total nicotine that is biologically available in the unprotonated form. The pH of e-cigarette aerosols varies from 4.85–9.6, whereas the pH of smoke from flue-cured tobaccos found in most cigarettes is acidic (pH 5.5–6.0). [43,70]. E-cigarette aerosols that are more alkaline (pH 6.5 or higher) results in nicotine existing primarily in the free-base form (unprotonated) which crosses the cell membrane for more rapid absorption. The long-term effect of exposure to-e-cigarette aerosols with a high pH is not known but might suggest that users will get their daily intake of nicotine faster than tobacco smokers, leading thus to reduction in vaping frequency and exposure to aerosols. Below, we further compare and contrast cigarette smoke toxicology with that of emerging data from e-cigarette aerosols.

### 6.1. Ultra Fine Particles (UFP)

PM can be classified according to their size: PM_10_: coarse particles less than 10 µm in diameter; PM_2.5_: fine particles less than 2.5 µm; and PM_0.1_: ultrafine particles (UFPs) smaller than 100 nm. Of these, PM_0.1_ has the potential to exert significant harm, as particles of this size can escape broncho-mucociliary action and scavenging by alveolar macrophages. These particles also penetrate deep into the respiratory tract (i.e., the alveolus) where they can be absorbed by the blood stream [71]. Smoking one cigarette exposes the human respiratory tract to between 10,000 and 40,000 μg PM (≈10^12^ particles per cigarette) [72] with a mean diameter <1 μm. Some studies indicate that e-cigarette aerosols contain less PM than cigarette smoke [73]. On the contrary, other studies state these aerosols contains high levels of UFP [74], reaching values of more than 2 × 10^5^ particles/cm^3^ [75]. Such discrepancies can be due to differences in the parameters used during the study (e.g., type of e-cigarette, brand, flavor, and voltage). However, PM from e-cigarettes evaporate within 10–20 s, and immediately after the vaping period, the aerosol concentration reaches initial background levels. On the contrary, PM emitted from cigarette smoke was lower in concentration but had a much longer lifetime (1.4 h). It was suggested therefore that the majority of e-cigarette aerosols are composed of volatile material, probably PG and/or VG [76].

The size and level of particulates in e-cigarette vapors have a bimodal particle size distribution of UFPs and submicron particles (96–175 nm) [77], with power and coil resistance greatly affecting e-cigarette aerosol count and mass distribution of particles [78]. This differs in comparison to that of a typical combustion cigarette, as the generation of particles in e-cigarette aerosols is smaller than those found in the smoke of combustible cigarettes [79]. This can be of concern as UFP can deposit deep in the lung and long-term exposure to UFP is associated with chronic inflammatory diseases such as COPD [71]. A dosimetry study estimated that an average of 6.25 × 10^10^ particles is deposited in the pulmonary tree after a single puff from an e-cigarette, with the highest deposition densities found in the lobar bronchi [80]. The effects of particles found in e-cigarette aerosols on the lung function remains unknown although PM and UFP are associated with cancer, cardiovascular, and respiratory diseases and cause epigenetic modifications, including alterations in the expression of noncoding RNAs. Such epigenetics changes can lead to dysregulation in genes expression [71,81]. Future studies should focus on identifying the nature of inhaled UFP from e-cigarettes and their negative impact on health.

### 6.2. Aldehydes

Formaldehyde, acetaldehyde, and acrolein are three toxic low-molecular weight aldehydes present in cigarette smoke (700–800 μg/cigarette in mainstream smoke) as well as e-cigarette aerosols (8.2 to 40.4 μg/10 puffs) [82]. While the concentration of aldehydes in e-cigarette aerosols depends on the voltage of the battery and the temperature of the heating-coil, the quantity of these is variable between devices. However, studies suggest that the level of aldehydes in e-cigarette aerosols can approach those from traditional cigarettes if the e-cigarette device is used at higher power settings (i.e., 5.0 V and more) or during dry puff conditions. Dry puff occurs when the atomizer heats up but does not have enough e-liquid to vaporize. In this case, the levels of aldehydes can increase up to 344.6 µg for formaldehyde and 206.3 µg for acetaldehyde [83]. Noteworthy is findings that there is production of carbonyls from thermal decomposition of PEG 400 and MCT. Also when heated to 230 °C, PEG 400 produced formaldehyde and acetaldehyde, two carcinogenic compounds, at levels that exceeded those produced by PG. PEG 400 and PG produced as much as 1.12% of the daily exposure limit/one inhalation, nearly the same exposure as smoking one cigarette. MCT and VG also produced low levels of aldehydes (approximately 33 times less than PEG 400) [84].

### 6.3. PAHs

PAHs are organic compounds composed of multiple aromatic rings that contain mainly carbon and hydrogen. PAHs are produced from the incomplete combustion of organic compounds and thus are present in smoke from forest fires, wood, and cigarettes. Exposure to PAHs activates the aryl hydrocarbon receptor (AhR) which induces the expression of xenobiotic metabolizing enzymes (XMEs). These XMEs, such as cytochrome P450 1A1 and 1B1 (CYP1A1 and CYP1B1, respectively) are important in the metabolism and clearance of most PAHs [85]. Many PAHs are toxic and/or carcinogens. For example, naphthalene is one of the most abundant PAHs (average intake rate by inhalation is 19 μg/day) [86] and is a respiratory toxicant as well as a possible human carcinogen (group 2B) [87]. In cigarette smoke, more than 500 PAHs are generated [88], and findings in animal models indicate that deleterious health effects are mediated by the AhR [85]. Benzo[*a*]pyrene (B[*a*]P) is another PAH present in cigarette smoke (<10 ng/cigarette) and is a group 1 carcinogen [89]. The type and amount of PAHs in e-cigarette aerosol is less than that of tobacco smoke [90]. In one study, Margham et al., examined the presence of 16 PAHs in different e-cigarette aerosols including dibenz[a,e]pyrene, naphthalene, and chrysene; the levels of these PAHs were 99.7% lower in e-cigarette aerosol compared to smoke from research cigarettes [91]. Overall, this suggests that e-cigarettes may pose less risk than tobacco because of the reduction in exposure to PAHs. Despite this, the presence of some of these PAHs in e-cigarette aerosols may also mean that vaping is not completely risk free.

### 6.4. Metals

E-liquids can contain traces of many inorganic elements and toxic metals such as sodium, bromine, gold, scandium, iron, and cobalt. The concentrations are much less than the respective ones in cigarette smoke, and thus, the risk associated with exposures could be very low. However, long-term studies are required to evaluate if these metals can accumulate in the lung and cause adverse effect after long-periods of exposure [92]. Moreover, in e-cigarettes, heating coils are usually made of nichrome (combination of nickel (Ni) and chromium (Cr)) and stainless steel. Toxic metals from heated coils can leach into vaping aerosols [91] and is the reason why Ni and Cr are present in e-cigarette aerosols but not the e-liquids [93,94]. This suggests that the Cr and Ni emission are higher than smoke from tobacco cigarettes. Analysis of cigarette smoke showed that emission levels were not quantifiable. However, Cr and Ni levels in e-cigarette aerosol were 50 ng in the first 100 puffs [91]. Ni and Cr are toxic for human and classified as a group 1 carcinogen by the International Agency for Research on Cancer (IARC); inhalation of these metals is associated with chronic bronchitis and reduced lung function [95]. In addition to Ni and Cr, copper and zinc are also detected in e-cigarette aerosols (0.2 µg generated from 100 puffs). Cadmium (Cd), a metal present in tobacco, is also found in aerosols but not in e-liquids. Analysis of four different e-cigarettes showed that the concentration of Cd in the e-cigarette aerosol is lower than in tobacco smoke (0.002 µg/15 puffs vs 0.056 µg/15 puffs, respectively) [96]. Cadmium accumulates in the lung of smokers, and although there is an association between Cd exposure and an increased risk of lung cancer, studies are inconclusive due to confounding factors such as the presence of other metals [97].

### 6.5. Other Toxicants

Aerosols generated from e-cigarettes also contain TSNAs and volatile organic compounds (VOCs) [10,43,45]. Among the VOCs found in e-cigarette aerosols, benzene is a main one, being classified as a known human carcinogen. Benzene is typically found in the air due to emissions from burning oil and motor vehicle exhaust. Cigarette smoke is also a major source for benzene, accounting for nearly half of all human exposures. A comparative study evaluated the emission of benzene in e-cigarette aerosols. In this study, benzene was not detected in JUUL. However, in two refill tank systems, benzene was formed from solvents (PG and glycerol) and additives (benzoic acid and benzaldehyde). Levels of benzene ranged between 1.8 μg/m^3^ and 5000 μg/m^3^, depending on the power settings and additives. Despite being much less than what is observed in traditional cigarettes (200,000 μg/m^3^), the concentrations of benzene found in e-cigarette aerosol still raises concern, especially considering that chronic, repeated exposure to benzene from e-cigarette aerosols might not be of negligible risk [62].

TSNAs are also present in trace amounts in e-cigarette aerosols. TSNAs are among the most important carcinogens in cigarette smoke and are formed during the curing process from nicotine and other tobacco alkaloids [98]. Total levels of TSNAs typically range from 200 to 1600 ng/cigarette [99]. Among the seven present in tobacco smoke, nicotine-derived nitrosamine ketone (NNK) and *N*-nitrosonornicotine (NNN) are regarded as the most carcinogenic [100]. Other TSNAs include *N*′-nitrosoanatabine (NAT) and *N*-nitrosoanabasine (NAB) [101]. Several studies have reported that certain TSNA have been detected in e-cigarette aerosols, but the levels are considerably lower than in tobacco cigarettes (0.8 ng to 28.3 ng/e-cigarette aerosols (150 puffs) [100]. For this reason, the FDA recently announced its intention to regulate TSNAs in e-cigarettes [100]. With the rise of vaping as alternative of smoking, it is important to monitor the levels of TSNAs in the body as a result of the use of e-cigarettes. Additional studies on the potential risk of these TSNAs would enhance our understanding the health concerns of long-term exposure to TSNAs from e-cigarette aerosols.

## 7. Cellular Alterations from E-cigarette Exposure

Toxicity caused by e-liquids and the resultant aerosol that is generated may be the result of numerous factors including the composition of the e-liquid, the temperature, dose, duration, and the cell type. Cells within the respiratory epithelium represent the first line of defense against pathogens and inhaled toxicants, including those in cigarette smoke and e-cigarette aerosols. The lower respiratory tract (from the trachea through to the alveoli) is lined with more than 50 different cell types, most of which are epithelial [102]. Approximately half of the epithelial cells are ciliated and interspersed with basal cells, goblet cells (which produce mucin), and Clara cells, a non-ciliated secretory cell that produces Clara cell secretory protein (CCSP). The gas-exchanging alveoli consist of an epithelial layer and extracellular matrix surrounded by capillaries. There are three major types of cells within the alveoli: type I pneumocytes which play role in gas exchange; type II pneumocytes which release pulmonary surfactants to lower surface tension; and alveolar macrophages, phagocytic cells that play major role in host defense [102,103]. Epithelial injury can serve as an initiating factor for a variety of lung diseases such as cancer and COPD. It is well known that smoking reduces the integrity of the epithelium barrier, thereby increasing the permeability of the respiratory epithelium, and impair host defense to reduce bacterial clearance. Moreover, cigarette smoke incites lung inflammation, induces oxidative stress, and causes DNA damage [104]. Studies are now emerging that indicate e-cigarettes also decrease cell integrity and induce inflammation, two effects that may be independent of the type of vaping device, cell type, as well as the presence of flavors and other components of e-liquids (e.g., solvents and nicotine) [102,105,106,107,108].

### 7.1. Inflammation

Chronic inflammation is thought to drive the development and progression of lung cancer and COPD [109]. Exposure to cigarette smoke causes airway inflammation and activates a molecular signaling cascade resulting in the production of cytokines and chemokines such as interleukin (IL)-1β, IL-6, tumor necrosis factor-α (TNF-α), monocyte chemoattractant protein-1 (MCP-1), and IL-8 [110]. Cigarette smoke increases permeability of the respiratory epithelium and causes mucous overproduction. This pro-inflammatory milieu in the lung leads to consecutive recruitment of immune cells. Neutrophils and macrophages are among the first cells recruited, but other immune cells such CD8^+^ T cells are also increased. Accumulation of these immune cells leads to further release of pro-inflammatory cytokines, chemotactic factors, reactive oxygen species (ROS), and proteases, thereby perpetuating this inflammatory response [110,111]. The inflammation-promoting effects of tobacco smoke inhalation are undisputed, and emerging evidence indicates that e-cigarette aerosols also promote inflammation in the respiratory system, albeit at lower levels. For instance, mice exposed to tobacco flavored e-cigarette aerosols containing nicotine had an increase in pro-inflammatory cytokine and chemokine secretion [112] as well as increased infiltration of neutrophils and macrophages [113]. Similar to cigarette smoke, after a 3-day exposure to e-cigarette aerosols, there were higher levels of Muc5ac [113], a predominant gel-forming mucin that is induced during allergy, and it also increased in the airways of smokers and e-cigarette users [114]. In addition, the levels of neutrophil extracellular traps (NETs) and the neutrophilic enzymes elastase and matrix metalloproteinase-9 (MMP-9) that are associated with the development of COPD, are significantly elevated in e-cigarette users [114].

One variable that may impact the potential adverse effects of e-cigarettes is the device settings. Users tend to increase heating element parameters by regulating battery voltage and electrical resistance of the coil. Indeed, a survey study on 522 adults showed that, on average, the user sets the power of their devices to 28.3 ± 24.2 W [115]. These variables as well as puff topography (e.g., puff duration, inter-puff interval, and number of puffs) affect aerosol generation and nicotine delivery and thus could impact the potential health effects of e-cigarettes, including the level of inflammation. For example, PG and glycerol aerosols generated by an e-cigarette device operating at more than 40 W (considered to be high wattage) induced the release of IL-6 and IL-8 by human bronchial epithelial [116]. In a comparative study, Cirillo. S. et al. used two identical devices containing the same e-liquid (PG/VG ratio, nicotine concentration, and flavors) but equipped with two different coils (1.5 and 0.25 ohm) to obtain total wattages of 8 ± 2 W and 40 ± 5 W, respectively. Aerosols generated at higher wattage induced a much more robust inflammatory response in rats compared to aerosols generated at lower wattage [117], suggesting that device characteristics are key factors that affect inflammation induced by e-cigarette aerosols.

Although in vitro and animal studies indicate that there are respiratory irritants in e-cigarette liquids capable of eliciting pulmonary inflammation, especially at high wattage, other parameters such as the fraction of exhaled nitric oxide (FeNO), a noninvasive marker of airway inflammation, and serum C-reactive protein (CRP), a nonspecific marker of systemic inflammation, are minimally affected by exposure to e-cigarettes [118,119]. Nitric oxide (NO) is a gas produced by many inflammatory cells due to the enzymatic activity of inducible nitric oxide synthase (iNOS). NO plays an important role as an antiviral molecule [120] and reduces the activity of macrophages, T lymphocytes, dendritic cells, mast cells, neutrophils, and natural killer (NK) cells [121]. FeNO is a test that measures the level of NO in parts per billion (PPB) in the exhaled air from the lung, is considered a marker of airway inflammation, and therefore is often used to determine the level of inflammation in allergic and eosinophilic asthma patients [122]. The extent to which NO is altered by e-cigarette exposure is controversial. Although individuals exposed to e-cigarettes had reduced FeNO immediately post vaping, which implies a reduction of lung inflammation [118], a separate study by Boulay et al. found no significant difference in FeNO or serum CRP after exposure to a laboratory made mixture of PG and glycerin [119].

Some effects may be due to nicotine itself. Interestingly, nicotine can both promote and reduce inflammation. By acting through nAChRs on immune cells, nicotine inhibits the function of the transcription factor nuclear factor-κβ (NF-κB) by increasing the phosphorylation of signal transducer and activator of transcription 3 (STAT3) [123,124]. Conversely, nicotine can activate immune cells by increasing their chemotactic activity, migration, and interaction with endothelial cells, resulting in a heightened inflammatory response [125]. Thus, numerous studies demonstrate that vaping causes an inflammation that is similar to that caused by cigarette smoke inhalation but probably to less of an extent. However, to be able to make definitive conclusions, more studies are required to better characterize the inflammatory response in the lung after long-term exposure to e-cigarettes.

### 7.2. Oxidative Stress

The pro-inflammatory effects of e-liquid aerosols may be mediated, at least partially, by ROS generation. ROS are chemically reactive species that contain a radical oxygen such as superoxide anion (O_2_^−^), hypochlorite (ClO^−^), peroxynitrite (ONOO^−^), and hydroxyl (^•^OH) or a non-radical oxygen such as hydrogen peroxide (H_2_O_2_). In the cell, ROS are generated during mitochondrial oxidative metabolism and serve as important signaling molecules in cell proliferation and survival. ROS are also produced by the cells in response to xenobiotics, damage, and infections (mostly by neutrophils and macrophages) as a mechanism of defense [126,127]. Excessive ROS levels leads to oxidative stress, which is defined as an imbalance between the production of ROS and their elimination by antioxidants enzymes (e.g., superoxide dismutase (SOD) and glutathione peroxidase (GPX)). In cigarette smoke, ROS are produced during the combustion process (120–150 nmol of ROS) [127]. Characterization of ROS in e-cigarette aerosols is more limited, and results are often contradictory. For instance, a recent study reported that ROS levels varies between 1.2–8.9 nmol/puff (with H_2_O_2_ accounting for 12–68% of total ROS) and that the amount ROS is dependent on the e-cigarette brand, flavor, and puffing regime. Moreover, ROS production increased eight times as the voltage increased from the 3.7 V to 5.7 V [128]. In addition, cigarette smoke and e-cigarette aerosols also induce ROS production by the cells themselves. Different e-cigarettes and JUUL pod flavors generated significant amounts of cellular ROS and mitochondrial superoxide production in bronchial epithelial cells and monocytes, resulting in increased inflammatory mediators such as IL-6, IL-8, and prostaglandin E_2_ (PGE_2_) [46,52]. Moreover, e-cigarette aerosol-induced ROS is capable of triggering apoptosis and programmed necrosis that reduces overall cell viability [129]. Other cell death machinery is perturbed by e-cigarette aerosols including autophagy, a process by which proteins and other cellular components are recycled in order to maintain cellular homeostasis. Autophagy is induced in response to cellular stress such as nutrient deprivation, oxidative stress, DNA damage, protein aggregates, damaged organelles, or pathogens, whereby it functions as a cytoprotective response [130]. E-cigarette exposure, similar to cigarette smoke, induces autophagy in human bronchial epithelial cells and murine lungs [131]. Nicotine inhalation was also linked with reduced cell viability via induction of cellular apoptosis/senescence through ROS-mediated autophagy [132]. Aldehydes also cause release of proinflammatory cytokines, proteases, and ROS in pulmonary and endothelial cells. These findings are of potential importance, as emphysema, the component of COPD characterized by alveolar loss, is mechanistically attributed to aberrant activation of these various cell death pathways [133]. In conclusion, vaping can induce cellular responses similar to those caused by smoking (e.g., oxidative stress and cell death), which are mechanistically linked to emphysema.

### 7.3. DNA Damage

Lung cancer is a leading cause of preventable death in the world, and the role of cigarette smoke in the etiology of lung cancer is well established. Many chemicals present in cigarette smoke such as the aldehydes and PAHs induce DNA adducts [134]. Several studies suggest that exposure to e-cigarettes also induces DNA damage [46,135,136,137,138], possibly because of increased oxidative stress [137,139]. However, the extent to which e-cigarette aerosols cause DNA damage is incongruent, with at least one study reporting that e-cigarette aerosols do not induce damage as measured by assessment of DNA double-stranded breaks [140]. This discrepancy may arise from differences in e-cigarette products and/or methodology (e.g., technique used to evaluate DNA damage, cell type, and/or method for generating the aerosol). E-liquids—as well as nicotine alone—may inhibit DNA repair mechanisms. At least five major DNA repair mechanisms exist, including base excision repair (BER), nucleotide excision repair (NER), mismatch repair (MMR), homologous recombination (HR), and nonhomologous end joining (NHEJ) [141]. Current evidence exists that e-cigarette aerosols impair DNA repair by reducing the level of xeroderma pigmentosum C (XPC) and 8-Oxoguanine glycosylase (OGG1/2) involved in NER and BER, respectively [112,136]. While emerging cell-based evidence indicates the possibility of DNA damage, these same data add to the difficulty in drawing conclusions and highlight the need for further studies to assess the link between long-term use of e-cigarettes and cancer.

### 7.4. Host Defense

Traditional cigarette smoke exposure increases susceptibility to influenza and other respiratory infections such as tuberculosis and pneumonia [142,143] through numerous effects on host defense mechanisms. Smoking impacts both innate and adaptive immunity. Innate immunity is nonspecific defense mechanisms including physical barriers (e.g., epithelium of the skin and lungs) and various immune cells such as macrophages and NK cells. Macrophages are phagocytic cells that engulf and digest cellular debris along with foreign substances such as microbes. Exposure to smoke inhibits the phagocytotic function of macrophages [144,145,146]. The cytolytic capacity NK cells, for which the primary function is to destroy virus-infected cells and to limit the spread of tumors, is attenuated by cigarette smoke [147]. Similarly, there is growing evidence that e-cigarette exposure decreases host defense mechanisms [51,107,148,149] by decreasing the phagocytic ability of macrophages, resulting in reduced bacterial clearance [142,149]. Moreover, e-liquid vapors decrease the production of the antiviral protein SPLUNC1 by epithelial cells and thus increase susceptibility to infection by rhinovirus, a respiratory virus that is the primary cause of the common cold [51]. Moreover, long-term exposure (3 months) of mice to e-cigarettes downregulates the antiviral immune response (secretion of interferon γ (IFN-γ)) by lung-resident macrophages against influenza virus [150]. Influenza is an acute respiratory infection associated with a significant morbidity and mortality. Influenza affects 10–20% of patients annually in developed countries, and there is increased susceptibility among smokers.

Adaptive immunity, defined by the presence of B and T lymphocytes, is also negatively affected by cigarette smoke, as numerous studies have shown that smoking increases the number of CD8+ T cells which may lead to emphysematous lung destruction [146]. There are currently no experimental studies on the effect of vaping on adaptative immune function in response to infections. The sole report in humans indicates that vaping decreases the expression of immune genes in nasal scrape biopsies including chemokine (C-X-C Motif) ligand 2 (CXCL2), chemokine (C-X3-C Motif) receptor 1 (CX3CR1), as well as cluster of differentiation 28 (CD28). [151]. CD28 is co-stimulatory signal required for T-cell activation, and the chemokines and their receptors play a critical role in the activation and recruitment of immune cells to sites of infection and inflammation [152,153]. Overall, these studies suggest that e-cigarettes can disturb the immune response, and future studies to address whether use of e-cigarettes increase the susceptibility to infections are needed.

### 7.5. Epigenetic Modifications

Epigenetics refers to changes in gene expression that do not involve changes in the DNA sequence and includes histone modifications, microRNA (miRNAs) and long noncoding RNAs (lncRNAs), and DNA methylation [71]. Cigarette smoke is now well established to cause epigenetic modifications. Smoke-induced DNA methylation is a possible mechanism behind smoke-induced diseases such as cancer. DNA methylation is also associated with vaping, with in utero e-cigarette exposure of mice leading to abnormalities in DNA methylation that was associated with a higher abnormal inflammatory environment in the lung of both the mothers and offspring [154]. Cigarette smoke also induces histone modifications and changes miRNAs levels in vitro as well as in vivo after smoke exposure and in individuals with COPD [155,156,157,158]. miRNAs are small noncoding RNAs that function in posttranscriptional regulation of genes expression by silencing mRNA expression, and their dysregulation is implicated in a number of smoke-related diseases [159,160]. There is now evidence that e-cigarette exposure alters the expression of 578 miRNAs in human lung epithelial cells, although the functional significance of these miRNAs and their contribution in the cytoxicity of e-cigarettes remains unknown [161]. Cigarette smoke also alters lncRNA expression such as Hox transcript antisense intergenic RNA (HOTAIR), colon cancer-associated transcript-1 (CCAT1), and metastasis associated in lung adenocarcinoma transcript 1 (MALAT1). LncRNAs are transcripts with lengths exceeding 200 nucleotides that are not translated into protein but function to control transcription and posttranscriptional mRNA processing. Similar to miRNAs, these functions often involve complementary base pairing with the target mRNA [162]. There is currently little information on e-cigarettes and lncRNA, although a recent report indicates that exposure to e-cigarettes alters the expression of long noncoding, antisense, small nucleolar, and miscRNAs [163]. Thus, it is possible that e-cigarettes regulate the expression of numerous genes partially through epigenetic mechanisms.

## 8. Clinical Impact of E-Cigarette Use on the Respiratory System

Although there is an existing body literature that suggests e-cigarettes could have a role in smoking cessation or reduction (reviewed in Section 3), the long-term health effects of vaping remain largely unknown. Furthermore, e-cigarette use by a previous never smoker may not be without harm, particularly if initiation occurs at a young age, given that young individuals have a longer time to accrue disease, or in individuals with existing pulmonary comorbidities. Available data summarized above indicates that e-cigarette use is associated with adverse cellular events that could lead to pulmonary alterations. Of concern are the increasing number of studies, presented below, showing that chronic e-cigarette use can have adverse clinical effects that are both similar yet different when compared to traditional cigarettes. As e-cigarettes are a relatively new product, there is uncertainity about the overall health effects of vaping. This lack of consensus is due to many factors, including the rapidly evolving e-cigarette technology, diversity of e-liquid composition, and the absence of standardization in e-cigarette products, all of which makes it challenging to compare results across studies. This is unlike traditional cigarettes, where the health consequences are well known and manufactured products are relatively uniform. There is however, a general agreement among many from the public health community, such as the Canadian Cancer Society and Canadian Thoracic Society, that while e-cigarettes could be helpful for smoking cessation in some individuals, they are not without harm [164,165]. From toxicologic analysis, risks are lower than those of traditional tobacco smoke, but the clinical impact is not known for individuals continuing to use e-cigarettes for the long term.

Complicating the ability to draw conclusions regarding the health effects of e-cigarettes is the discrepancy between counterfeit products, which vary significantly in composition of liquid (inclusion of cannabis derivatives and/or flavoring agents associated with pulmonary toxicity) and hardware (e.g., type of heating coil). Case reports of acute lung diseases have been plenty over the past decade, leading up to the recognition of a new clinical entity in 2019 now referred to as EVALI. A CDC lead investigation has shown that the majority of EVALI cases were associated with use of counterfeit products [166]. As such, the long-term clinical impact of e-cigarettes will likely be related to many different factors, including age of initiation, current and prior cigarette smoking status, and the presence of preexisting lung conditions such as asthma and COPD. The capacity of industry and regulatory bodies to standardize these products will also be a factor. Although e-cigarettes differ from tobacco cigarettes in that there is substantially less second-hand emissions [167], the clinical impact of second-hand e-cigarette emissions to the nonuser also remains uncertain.

### 8.1. Respiratory Symptoms

Multiple respiratory tract symptoms, including acute cough, sore throat, and dry mouth have been reported after use of e-cigarettes [168]. Among youth, four studies that examined respiratory symptoms were cross-sectional with user self-reported questionnaires, and provided evidence for an association between respiratory symptoms and e-cigarette use. There is a significant association between chronic bronchitis symptoms (daily cough for 3 months in a row, congestion, or phlegm production other than accompanied by a cold in the preceding 12 months) and e-cigarette use [154,169] as well as a self-reported clinical diagnosis of asthma [170]. In addition, employees from the entertainment industry chronically exposed to glycerin and mineral oil-containing fog machines reported more chronic work-related wheezing and chest tightness [171]. Nonetheless, in contrast to previous studies, Bouley et al. report that both healthy and asthmatic volunteers had no difference in cough, chest tightness, breathlessness, respiratory secretions, and wheezing after acute exposure, e.g., 1 h session of vaping, to a laboratory made mixture of PG:VG [119]. This discrepancy likely reflects the short, single exposure in a controlled laboratory setting which is in sharp contrast to the chronic, multivariable conditions of real-life use. Studies in animal models suggest that PG inhalation has limited biological effects and no signs of toxicity [172], with a no-observed adverse effect level (NOAEL) of 1000 mg/m^3^ for aerosolized glycerol [173]. Collectively, these observations whereby e-cigarette use is associated with chronic bronchitis symptoms in adolescence is potentially concerning, as a more rapid decline in lung function in later life has been linked to asthma and chronic bronchitis in early life.

### 8.2. Airway Mechanics (Lung Function)

Airway mechanics are used to characterize lung function by evaluating pressure and airflow at different stages of the respiratory cycle; alterations in respiratory mechanics is indicative of lung dysfunction and/or disease development/progression. Commonly used metrics include flow volumes during expiration, flow rate during expiration, lung volumes at various points in the respiratory cycle, static airway resistance, and compliance (or its inverse—elastance) of the lung and chest wall. These measures are usually taken using spirometry and body plethysmography. A decrease in lung elastance (or increased lung compliance) and/or increased resistance causes a reduction in flow volumes and flow rates during expiration. This is manifested by a decline in the FEV_1_ (fraction of expired volume in 1 s), the forced vital capacity (FVC), and PEF (peak expiratory flow). FVC is the total volume of air that a person can expire during a full forced exhalation, whereas FEV_1_ is the proportion of that volume that is exhaled in the first second of expiration. Healthy subjects normally breathe out at least 70% of this volume within the first second. A percentage less that this is usually consistent with an obstructive lung disease such as COPD or asthma. When compared to cigarette smoking, the consequences of vaping on lung function are not well studied. However, available data indicates that there are no significant changes after e-cigarette use in FEV_1_, FVC, or PEF. For example, Flouris et al. compared pulmonary function before and after a session of e-cigarette use in both smokers and nonsmokers. Although there was no difference in FEV1, FVC, or PEF, there was an increase in respiratory impedance [174]. Respiratory impedance is an indicator of airway resistance when measured by impulse oscillometry system (IOS). IOS is a noninvasive mechanism that can identify changes in airway obstruction and resistance earlier in the disease process when compared to conventional spirometry and/or methacholine challenge [175]. This indicates that vaping may induce early subclinical changes in respiratory function. However, at this time, there is a lack of well-designed epidemiological studies examining whether there are long-term effects of cigarette usage on lung function.

### 8.3. Acute Pulmonary Disease

Acute pulmonary diseases associated with e-cigarette use have garnered significant attention recently due to multiple case reports linking the two together. The large majority of these cases fall under the category of EVALI (discussed below), which has been largely attributed to the use of counterfeit vaping products [39]. The safety of e-cigarettes is inconclusive, and there are studies pointing to their safety. In one such study led by Fontem Ventures (a subsidiary of Imperial Brands and maker of blu), a two-year evaluation of one popular commercially available e-cigarette concluded that no acute respiratory events were noted [176]. However, a number of acute respiratory events that fall outside the definition of EVALI have also been reported; these include spontaneous pneumothoraces, acute eosinophilic pneumonia, and bronchiolitis obliterans. Some of these acute pulmonary diseases (primary spontaneous pneumonthorax and acute eosinophilic pneumonia being notable exceptions) have also been reported with combustible cigarette smoking.

#### 8.3.1. Primary Spontaneous Pneumothorax (PSP)

A pneumothorax refers to the presence of air in the pleural space lining the lungs. Pneumothoraces can be classified as traumatic, iatrogenic, or spontaneous. Spontaneous pneumothoraces are those that occur without an inciting external event such as trauma or a medical procedure. Spontaneous pneumothoraces are commonly seen as a secondary spontaneous pneumothoraces (SSP) due to underlying lung diseases but can also be primary (PSP). PSP is more common in men, and one study estimates the incidence of PSP to be 24.0 per 100,000 men per year and 9.8 per 100,000 women per year [177]. Recurrent PSPs have been reported in a Caucasian teenager who was a regular user of e-cigarettes without any other identified risk factor and/or cause [178]. While conventional cigarette use is a known risk factor of spontaneous pneumothoraces, spontaneous pneumothorax associated with e-cigarette use was novel. Treatment of pneumothoraces involves treating the underlying cause, if any, and evacuation of the air by a chest tube. In this case, the patient had presented with two separate pneumothoraces that required chest tube drainage within two weeks of each other [178].

#### 8.3.2. Acute Eosinophilic Pneumonia

Acute eosinophilic pneumonia is characterized by filling of the alveolar airspaces with eosinophil-rich infiltrate (as opposed to neutrophil-rich infiltrate seen in bacterial pneumonia). Acute eosinophilic pneumonia can be idiopathic or secondary to various medications, toxins, or parasitic infections. True idiopathic acute eosinophilic pneumonia is a rare disease entity and was first described in 1989 [179]. A number of case reports have now linked e-cigarette use and acute eosinophilic pneumonia. Cases usually present with acute respiratory symptoms and bilateral opacities on chest imaging and, unlike EVALI, presents with a BAL that is predominantly eosinophilic (between 25–60% in reported cases) [180]. Treatment of the acute eosinophilic pneumonia is with systemic glucocorticoids and supportive therapy until disease resolution.

#### 8.3.3. Bronchiolitis Obliterans

Bronchiolitis obliterans is a clinical syndrome caused by inflammation and obliteration of small airways. The syndrome comprises of dyspnea, irreversible airflow obstruction, and hyperinflated lungs. Known causes of bronchiolitis obliterans include inhaled toxicants, infections, drug exposures, or graft-versus-host disease after hematopoietic cell transplantation or lung transplantation. The treatment of bronchiolitis obliterans largely comprises symptom management and stopping any inciting agents. Various pharmacological agents—including macrolide antibiotics and glucocorticoids—have also been used with varying success. Some cases of bronchiolitis obliterans may eventually require lung transplantation. Bronchiolitis obliterans was dubbed “popcorn lung” in workers at a microwave popcorn production plant that was linked to inhalation of diacetyl (2,3-butanedione), which is commonly used to provide a creamy/buttery flavor [181,182]. Diaceyl is also present in e-cigarettes [183]. A case of bronchiolitis obliterans associated with e-cigarette use has been reported in Canada, although the authors were not able to verify the presence of diacetyl in the patient’s e-liquid [184].

#### 8.3.4. Vaping-Associated Lung Injury (EVALI)

There has been an outbreak of an acute respiratory illness associated with counterfeit vaping product use. In the summer of 2019, public health departments in the states of Wisconsin and Illinois in the United States noticed an uptick of acute respiratory illness in young individuals without any known apparent cause and with the only commonality being previous exposure to e-cigarettes. As of February 18, 2020, 2807 cases of EVALI have been reported across the United States. Sixty-seven percent of EVALI cases occurred in males; 78% were under the age of 35 years, with a median age of 24 years. Sixty-eight of these patients died [185]. In Canada, 14 cases of EVALI have been reported to the Public Health Agency of Canada. Seven were female, and 7 were male; 3 cases were between the ages of 15–19, 3 were between 20–34, 3 were between 40–49 years, and another 4 were over the age of 50 [186]. The largest case series of EVALI thus far is a report of 98 cases from the public health investigation in Wisconsin and Illinois. It indicated that 97% of cases presented with respiratory symptoms including shortness of breath, chest pain, cough, and hemoptysis [187]. While a more thorough case definition is available elsewhere, a “confirmed” case of EVALI consists of (1) use of an e-cigarette in the 90 days prior to symptom onset, (2) pulmonary infiltrates on chest x-ray and or CT of the chest, (3) absence of pulmonary infections on initial workup, and (4) no evidence of alternative plausible diagnosis [187].

##### Could EVALI Be an Exogenous Lipoid Pneumonia?

First described in 1925, lipoid pneumonia occurs when lipids (often from exogenous sources) enter the lungs and accumulate within the alveoli and airspaces. Exogenous lipoid pneumonia can be the result of aspiration or inhalation of petroleum-based products and is seen in “fire-eater” performers who inhale the petroleum-based fluid as part of their performance. It is not without precedent however that lipoid pneumonia could also be caused by inhaled agents. A link between lipoid pneumonia and smoking of blackfat tobacco was identified in 1968 [188]. Manufactured in Kentucky, blackfat is a tobacco leaf to which mineral oil and vaseline are added for flavouring and as humectants and was imported into Guyana from the 1870s [189]. It was suggested that, during smoking, the oily material in blackfat tobacco (12.6% lipid) distills into the lungs to cause lipid pneumonia [189,190].

Examination of the BAL fluid and biopsy specimens of patients with EVALI have been useful in this regard. One commonality that has been noted in BAL fluid is the presence of lipid-laden macrophages [191,192]. Additionally, a recent experimental study suggests that e-cigarettes (independent of nicotine) alter lipid homeostasis in a mouse model of chronic exposure; a prominent feature included accumulation of lipids in macrophages from exposed mice [150]. While lipid laden macrophages could indicate an exogenous lipoid pneumonia, their presence remains a nonspecific marker and can be seen in a wide variety of medical conditions, including chronic aspiration [193]. Furthermore, a review of 17 pathology samples collected from EVALI cases identified no pathologic features of exogenous lipoid pneumonia [8]. Instead, features of airway centric pneumonitis—with bronchiolitis, bronchial wall edema, and mucosal ulceration—in addition to the accumulation of lipid laden macrophages and cytoplasmic vacuolization were found [8]; this was theorized to be the result of exposure to e-liquids containing an exogenous lipid (glycerin) rather than exogenous lipoid pneumonia [8].

##### The Role of Vitamin E Acetate

At present, the likely culprit associated with EVALI has been identified as vitamin E acetate used in THC-containing, often counterfeit, vape products. A study by the Minnesota Public Health Department found that 24 products obtained from 11 out of 12 EVALI patients contained vitamin E Acetate, while no vitamin E acetate was found in 10 products collected in 2018 prior to the outbreak of EVALI [194]. In addition, vitamin E acetate was found in BAL fluid from 48 of 51 EVALI cases. In comparison, vitamin E acetate was not found in any BAL fluid from a comparison group of 99 individuals comprised of never smokers, exclusive nicotine-only e-cigarette smokers, and exclusive conventional cigarette smokers [195]. Finally, a recent experimental study found that exposure of mice to inhaled aerosols of vitamin E acetate showed the presence of lipid-laden macrophages within the lungs [196].

## 9. Chronic Pulmonary Disease

E-cigarette use is still a relatively new phenomenon, having become widely popular in the last decade. Consequently, strong associations—and causality—between e-cigarette use and chronic pulmonary diseases are yet to be established. Nonetheless, there is emerging data to suggest a link between e-cigarettes and chronic lung diseases such as asthma and COPD [197,198].

### 9.1. COPD

COPD is a chronic pulmonary disease characterized by irreversible expiratory airflow limitation and dynamic hyperinflation. COPD comprises multiple phenotypes—including emphysema and chronic bronchitis. In the emphysematous phenotype, the lung parenchyma is damaged, resulting in reduced lung elastance, while in chronic bronchitis, narrowed airways hinder expiratory airflow. COPD is an important cause of morbidity and mortality around the world. In 2010, it was the third most common cause of mortality [199]. The treatment of COPD is multimodal and includes inhaled bronchodilators, inhaled corticosteroids, patient education, pulmonary rehabilitation, smoking cessation, and long-term oxygen therapy. The association of COPD with cigarette smoking is well established, and approximately 80–90% of all COPD cases are caused by cigarette smoking [200]. Data collected from a large cross-sectional telephone survey of adults in the United States revealed increased odds of self-reported COPD among e-cigarette users, including never smokers. Similar results were found when comparing current e-cigarette users and never users who are current conventional cigarette smokers and who are former conventional cigarette smokers [198] and thus is suggestive of a relationship between e-cigarette use and COPD. As discussed in Section 7, vaping—like cigarette smoking—is also associated with numerous pathogenetic mechanisms believed to drive the development of COPD, including inflammation, oxidative stress, and apoptosis. Although available evidence has yet to definitely link e-cigarette use to COPD causation, preclinical studies support that chronic exposure of mice to e-cigarettes increases distal airspace enlargement (reminiscent of an emphysema phenotype) in a nicotine-dependent manner [201,202]. However, contrary to the previous study, Olfert et al. reported that mice chronically exposed to different flavors of nicotine-containing e-cigarettes did not develop an emphysematous phenotype—contrary to cigarette smoke-exposed mice [203]. These divergent reports make it difficult to conclude whether vaping causes direct lung damage.

Given the paramount importance of smoking cessation in the case management in COPD, e-cigarettes may play an important role, as smoking cessation reduces lung function decline in patients with COPD. There is some evidence of improvement in COPD smokers who switch to e-cigarettes [204]. However, the utility of e-cigarettes for smoking cessation in COPD may be limited for a number of reasons. First, as previously discussed, current evidence indicates that e-cigarettes are not as effective as currently available, well-recognized, and optimal pharmacotherapy. Second, the creation of dual users or smokers with reduced conventional cigarette use may not confer the same benefit as smokers who are able to achieve complete cessation. Third, population level evidence that e-cigarette users are more likely to self-report a diagnosis of COPD independent of their conventional cigarette use, indicating that e-cigarettes themselves are possibly not without risk in the development of chronic lung disease. There is a need for additional studies, including prospective cohort studies that assess respiratory health outcomes in e-cigarette users compared with combustible tobacco users and dual users. These studies could provide valuable information regarding the respiratory health effects of substituting, completely or partly, combustible cigarettes with e-cigarettes on current smokers and the impact of substitution on the progression of preexisting lung disease.

### 9.2. Asthma

Asthma is another common obstructive pulmonary disease characterized by chronic airway inflammation and variable expiratory airflow limitation, resulting in the characteristic symptoms of chest tightness, wheeze, and cough. There can be commonalities between asthma and COPD, but the most useful distinction between the two is that, in asthma, unlike COPD, the airflow is variable and reversible with bronchodilators. It is estimated that roughly 300 million people globally have asthma. The pathogenesis of asthma is the result of a complex interaction of genetic and environmental factors. Cigarette smoking, both active and passive, has been identified as an environmental risk factor associated with an increased incidence of asthma. A prospective cohort study of African American women found that the incidence of asthma was higher in current active smokers, former active smokers, and passive smokers compared to never smokers [205]. In terms of e-cigarette use, an analysis of data collected as part of the same large cross sectional telephone survey mentioned in Section 9.1 indicated that the odds of a self-reported diagnosis of asthma was higher among current e-cigarette users in comparison to never-users. Interestingly, the odds of self-reported asthma increased with e-cigarette use frequency and remained increased regardless of conventional cigarette use status [197]. A dearth of experimental evidence makes drawing conclusion on e-cigarette use and asthma difficult, but studies indicate that flavorings may cause a differential response. In one study, mice that were challenged with house dust mite (HDM) and exposed to cinnacide-flavored e-cigarette aerosol had reduced airway inflammation and increased airway hyperresponsiveness (AHR) whereas exposure to banana-flavored e-cigarette aerosols trended towards increased airway inflammation. In contrast, all e-cigarette aerosols containing nicotine suppressed airway inflammation but did not alter airway hyperresponsiveness (AHR), suggesting that both flavors and nicotine affects the outcome of vaping on allergy [206]. However, in another study, mice challenged with ovalbumin and that were exposed intratracheally to e-liquids had increased infiltration of inflammatory cells, including eosinophils; aggravated AHR; and induced the secretion of the TH2 cytokine IL-4, IL-5, and IL-13 [207]. It remains difficult to draw conclusions, as these studies used different allergens, flavors, protocol of exposure, and route of administration. Therefore, more studies are needed before we can conclude whether vaping modulates asthma symptoms.

## 10. Limitations

One of the limitations in extrapolating results from experimental studies towards human relevance is the pattern of e-cigarette use or vaping topography, which refers to how an e-cigarette is used and includes parameters such as puff duration, volume of puff, interpuff interval, duration of session, and frequency. Numerous studies have evaluated vaping topography and showed that it varries widely [208,209,210]. For example, puff duration as well puff interval were between 1.5–2.1 s and 176.7–382.7 s, respectively [208]. For this reason and in addition to the numerous e-cigarette devices/liquids, brands, and power settings (e.g., battery voltage and coil resistance), it can be difficult to draw conclusions regarding user exposure to e-cigarette aerosols, their toxicants, and overall health impact [208]. The increasing evidence from in vitro studies and animal models showing that e-cigarettes have adverse pulmonary effects is based on variable experimental approaches between studies and using different/unique regimes of exposure, only some of which mimic human exposures. Moreover, details of aerosol generation are often limited, with many studies failing to report details of coil resistance [113,131], applied power [112,131], and the regime of exposure [112,131]. However, in studies where the exposure regime in mice is close to that of the average user (puff: 2 s, volume of puff: 35 mL, and duration of exposure: 1.5 h) and where serum cotinine levels are comparable to those found in both cigarette smokers and e-cigarette users [211], there was induction of a modest lung inflammatory response. As discussed above and shown is a recent study [212], the extent to which lung inflammation is induced in humans by e-cigarette exposure is controversial. A combined approach using both animal models of vaping whereby the exposures mimic user topography as well as assessment in people who vape will assist in our long-term understanding of e-cigarette use and its effects on the respiratory system.

## 11. Conclusions

**Figure 1:**
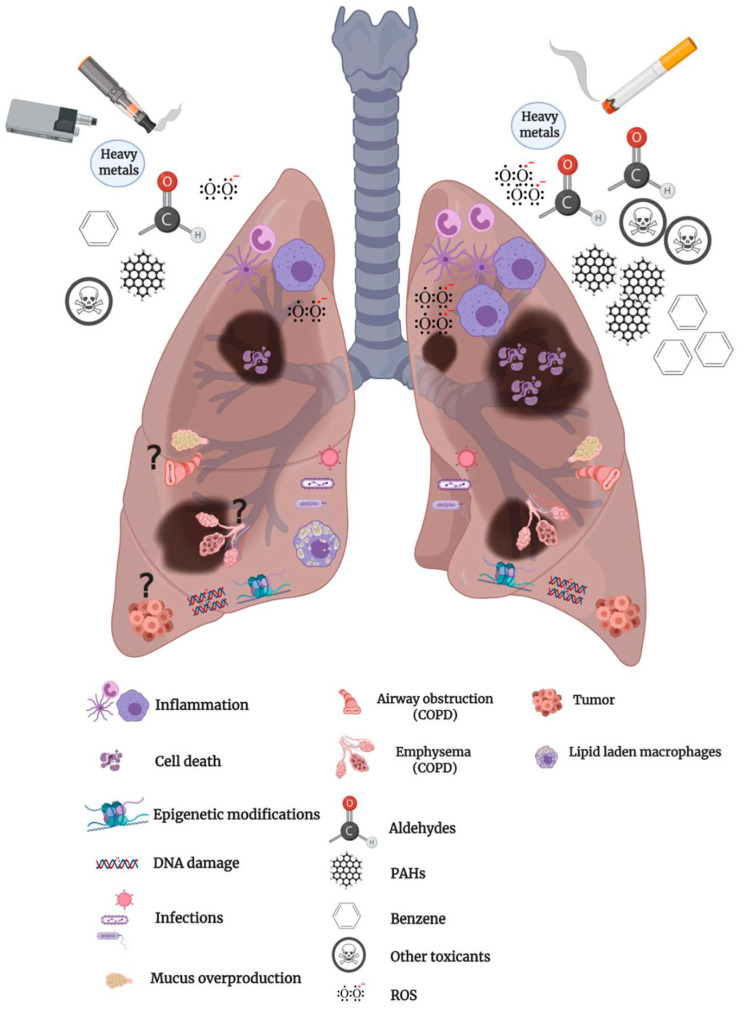
The known and unknown health effects of vaping in comparison to cigarette smoke. The major toxic effects of compounds found in cigarette smoke (Right lung) and in vaping aerosols (Left lung) are lunginflammation, oxidative stress, cell death, impaired immune response, DNA damage and epigeneticmodifications. The respiratory diseases caused by cigarette smoke (lung cancer, COPD [emphysema and/orobstruction of airways]) are not yet established to be caused by vaping (represented by question marks in theleft lung). The presence of lipid-laden macrophages is a feature predominantly associated with vaping products containing THC and has been a feature of EVALI.

E-cigarette use is rising rapidly among both smokers and nonsmokers. The chemical composition of the aerosol produced by e-cigarettes varies depending on parameters such as the device, voltage used, and the composition of e-liquid. Compared to tobacco smoke, many of the compounds found in e-cigarette aerosols are considered toxic or carcinogenic, including aldehydes, heavy metals, and TSNAs. Present studies suggest that even short-term e-cigarette use causes similar effects as tobacco smoke including cellular inflammation, apoptosis, oxidative stress, and DNA damage (Figure 1). These pathological processes are an important driver of many respiratory diseases such as COPD. Clearly, there is much about the effect of vaping that we do not know, including whether vaping causes decline in lung function similar to smoke, how vaping might lead to respiratory diseases, and which group of users are at highest risk? Although for current smokers e-cigarettes can be viewed as a “lesser of evils”, the effect of e-cigarette products on respiratory health may not be known for many years. Therefore, long-term epidemiological, toxicological, and clinical studies are required to build a more solid body of evidence, allowing us to reach more definitive conclusions on the potential harms of e-cigarette use. Until we know more on the effects of e-cigarettes on pulmonary health, we must take into account age, current and prior cigarette smoking, the presence of preexisting lung conditions such as asthma and COPD, and the potential for other pulmonary complications when considering the risk versus benefit equilibrium of e-cigarette use.

## Abbreviations

EVALI	vaping product-associated lung illness
COPD	chronic obstructive pulmonary disease
PG	propylene glycol
VG	vegetable glycerin
BAL	bronchoalveolar lavage
THC	tetrahydrocannabinol
CBD	cannabidiol
PM	particulate matter
GRAS	generally recognized as safe
PEG	polyethylene glycol
MCT	medium chain triglycerides
nAChRs	nicotinic acetylcholine receptors
TSNAs	tobacco-specific nitrosamines
PAHs	polycyclic aromatic hydrocarbons
UFP	ultrafine particles
miRNA	micro RNA
AhR	aryl hydrocarbon receptor
XMEs	xenobiotic metabolizing enzymes
VOCs	volatile organic compounds
FDA	food and drug administration
FeNO	fraction of exhaled nitric oxide
CRP	C-reactive protein
NO	nitric oxide
NK	natural killer cells
PPB	parts per billion
ROS	reactive oxygen species
H_2_O_2_	hydrogen peroxide
BER	base excision repair
NER	nucleotide excision repair
CD28	cluster of differentiation 28
FEV1	fraction of expired volume in 1 s
FVC	forced vital capacity
PEF	peak expiratory flow
OS	impulse oscillometry system
PSP	primary spontaneous pneumothorax
AHR	airway hyperresponsiveness
